# The effectiveness of retreatment with peginterferon alfa and ribavirin in patients with chronic viral hepatitis C genotype 2 and 3: a prospective cohort study in Brazil

**DOI:** 10.1186/1471-2334-12-377

**Published:** 2012-12-27

**Authors:** Simara Artico, Karine Medeiros Amaral, Candice Beatriz Treter Gonçalves, Paulo Dornelles Picon

**Affiliations:** 1 Universidade Federal do Rio Grande do Sul (UFRGS), Porto Alegre, Brazil; 2 Centro de Monitorização de Medicamentos Injetáveis (CAMMI), Hospital Sanatório Partenon, Porto Alegre, RS, Brazil; 3 Department of Internal Medicine, Universidade Federal do Rio Grande do Sul (UFRGS), Hospital de Clínicas de Porto Alegre (HCPA), Porto Alegre, Brazil; 4 Health Department of the State of Rio Grande do Sul, Porto Alegre, Brazil

**Keywords:** Hepatitis C, Retreatment, Peginterferon alpha, Ribavirin

## Abstract

**Background:**

More than 50% of patients infected with chronic hepatitis C virus (HCV) do not respond to treatment with conventional interferon (IFN) combined with ribavirin (RBV). The aim of our study was to evaluate the effectiveness of retreatment with peginterferon alfa-2a or 2b (PEG-IFN 2a or 2b) concomitantly with RBV in patients with HCV genotype 2 and 3, which were non-responders or relapsers to initial treatment with IFN / RBV and to identify possible predictors of sustained virological response (SVR).

**Methods:**

From September 2003 to March 2009 a cohort of 216 patients who had previously failed therapy with a regimen of standard interferon and ribavirin, were followed in a specialized service implemented in the Brazilian Unified Health System, Rio Grande do Sul. All patients were retreated with PEG-IFN 2a or 2b per week, associated with RBV, through oral route, with doses determined according to weight (1,000 mg if weight ≤ 75 Kg and 1,250 mg if ≥ 75 Kg) per day for 48 weeks. The HCV-RNA was tested by Polymerase Chain Reaction (PCR). Virological Response (VR) within 48 weeks and SVR in the 72 weeks was considered for evaluation of treatment efficacy. Analyses were performed in patients who received at least one dose of PEG-IFN.

**Results:**

The SVR rate for non-responders to previous treatment was 34.4% and for relapsers was 50% (p = 0.031). As predictive factors that contribute to improve SVR, were identified the age (p = 0.005), to be relapsers to previous treatment (p = 0.023) and present liver biopsy examination Metavir F0-F2 (p = 0.004). In assessing the safety profile, 51 patients (23.6%) discontinued treatment prematurely.

**Conclusions:**

This alternative retreatment for patients who have failed prior therapies for anti-HCV, has demonstrated promising SVR rate, provided that it includes a careful selection of patients with predictors of response and adverse events monitored.

## Background

Since identification of its causative agent in 1989, hepatitis C has been recognized as a major public health problem, with major economic and quality of life impact on peoples [1]. The hepatitis C virus (HCV) is currently the leading cause of chronic hepatitis and is a major cause of cirrhosis worldwide [2]. It is estimated that about 3% of world population are chronically infected by this agent, with at least six types of viral genotypes. In Brazil, are found mainly genotypes 1a, 1b, 2a, 2b and 3, with a predominance of genotype 1 over non-1 genotypes, with distribution of 60% and 40%, respectively. Among patients diagnosed in Brazil with non-1 genotype, approximately in 25% it is observed genotype 3 and 5% are diagnosed with genotype 2 [3].

Initially, the ideal treatment for chronic hepatitis C was the use of interferon alpha (IFN-α) monotherapy at a dose of 3 million units, thrice a week for 48 weeks, with rates of sustained virological response (SVR) of only 12 to 16% [4-8]. Shortly after, there are several clinical studies demonstrating the superiority of combination therapy with ribavirin (RBV) on IFN-α monotherapy and in 1998 the scheme is approved by the Food and Drug Administration (FDA) for treatment of chronic viral hepatitis C. Using this combined regimen for 24 or 48 weeks resulted in an increase of 2 to 3 times the SVR rates ranging from 36% to 47% [9-11].

More recently, the incorporation of an inert molecule of polyethylene glycol molecule to IFN produced a new drug to treat hepatitis C, peginterferon alfa (2a or 2b) with reduced kidney clearance (slower elimination), altered metabolism (more slow absorption) and increase in half-life, allowing his administration to become once a week instead of three times [12-14]. Using this new molecule has shown SVR rates of 54% to 56%, according to some clinical trials [15-17].

When the pegylated interferon was introduced in the market, a sizeable proportion of patients had not yet been successful with conventional interferon-based regimens. As chronic hepatitis C virus infection can result in long term complications (cirrhosis, terminal liver failure and hepatocellular carcinoma), those patients remain at risk of developing progressive liver disease and the possibility of an alternative to retreatment has been the focus of clinical investigations [18-20]. Literature data show that it has been achieved a global response to retreatment of 30-60% [21-32].

In Brazil, according to Chronic Viral Hepatitis C Therapeutic Guideline (CVH-PTG) - Ministerial Decree N°. 863 of November 4, 2002, the standard treatment of hepatitis C genotype 2 and 3 should be done with interferon alfa-2a (IFN-2a) or interferon alfa-2b (IFN-2b) at a dose of 3 million units, thrice a week subcutaneously combined with an oral dose ribavirin: 1,000 mg/day for patients under 75 kg and 1,250 mg/day, for those weighing over 75 Kg for 24 weeks. This drug is provided free by the Ministry of Health in capsules of 250 mg. For patients with hepatitis C genotype 1, with higher resistance to antiviral therapy, the recommended standard treatment is with peginterferon alfa-2a (PEG-IFN-2a) at a dose of 180 μg or peginterferon alfa-2b (PEG-IFN-2b) at a dose of 1.5 μg/Kg body weight once a week subcutaneously, associated with an oral dose ribavirin: 1,000 mg/day for patients under 75 kg and 1,250 mg/day, for those weighing over 75 Kg for 48 weeks [33]. On September 28, 2007, through the publication of Ordinance N°. 34, this Guideline was updated, including the use of PEG-IFN-2a or 2b as the standard treatment in the retreatment of patients genotypes 2 and 3 non-responders or relapsed after treatment with interferon alfa and ribavirin [34].

For the effective implementation of CVH-PTG, the State Health Secretariat of Rio Grande do Sul/Brazil established in Parthenon Sanatorium Hospital (PSH), the first Center for Application and Monitoring of Injectable Drugs (CAMID), a service aimed at the care of patients with hepatitis C. This service is aimed at improving the pharmaceutical assistance with the implementation of pharmaceutical care to administer and systematically monitor patients receiving PEG-IFN and ribavirin [35].

Therefore, the aim of this study was to evaluate the effectiveness of retreatment with peginterferon alfa-2a or 2b combined with ribavirin for 48 weeks in patients with chronic hepatitis C non-responders or relapsers to previous treatment with interferon alfa who were followed by a pharmaceutical care program of the State Department of Health of Rio Grande do Sul/Brazil and to identify possible predictors of SVR.

## Methods

### Design

All patients with chronic hepatitis C genotypes 2 and 3 were studied in a prospective cohort study in the CAMID - Parthenon Sanatorium Hospital from September 2003 to March 2009.

### Patient selection

All Two hundred and sixteen patients with chronic hepatitis C genotype 2 and 3 who underwent the first treatment for 24 weeks with interferon alfa-2a or 2b at a dose of 3 million units thrice a week subcutaneously and oral dose daily ribavirin (1,000 or 1,250 mg, depending on body weight). Out of these, 128 patients never achieved undetectable HCV RNA serum levels (qualitative PCR testing) during the first treatment (non-responders) and 88 patients showed undetectable HCV RNA during the first therapy but became HCV RNA positive after discontinuing medication (relapsers). All patients enrolled in the study who received at least one dose of medication were included in the statistical analysis. The study excluded all patients who did not agree to participate in interviews of monitoring during treatment.

### Sampling

Patients in the study were sequentially allocated according to demand.

### Follow-up of patients and data collection

The monitoring of patients was performed by a multidisciplinary team, with the presence of the pharmacist through monthly interviews. In these interviews, patients were informed about the disease, treatment and monitored for adverse events and laboratory tests needed for continuing care. The nursing staff performed the weekly applications of PEG-IFN, monthly monitoring body weight for possible dose adjustments where necessary.

Patient information and interventions were recorded on a specific Pharmacotherapeutics file, which contributed to the clinical and laboratory data such as prior treatment hepatitis C, virus C genotype, comorbidities, use of other medications, alcohol use, initial laboratory tests [albumin, prothrombin time, bilirubin, glucose, uric acid, ALT, AST, creatinine levels, TSH, hemoglobin and platelets]. An initial quantitative PCR and liver biopsy for assessing inflammatory activity and fibrosis grade were evaluated by the Metavir score. Patients were considered as carriers of non significant fibrosis when classified as F0, F1 and F2. Patients with F3 and F4 were considered as significant fibrosis carriers. that were recorded on the database developed especially for the service, allowing the pharmacoepidemiological study data [35].

As material to support the process of pharmaceutical care was used a "Patient Orientation Guide" containing information that was conveyed orally and in writing to the patient [35].

Adverse events presented by the patients were collected in a systematic way of monthly interviews with structured questions in advance, first questioning the patient about his condition and then about possible previously reported effects. The events were also classified according to intensity as mild, moderate or severe.

According to the protocol of the Ministry of Health, the quantitative PCR test at 12 weeks or 24 weeks in the qualitative PCR determined whether to proceed the treatment. Those patients who did not decrease by at least 2 log the viral load became negative or had their treatment suspended. Treatment response was evaluated by qualitative PCR tests of the end of treatment (48 weeks Virological Response - VR) and 24 weeks after its completion (72 weeks Sustained Virological Response - SVR) [33].

The interventions performed during the monitoring of treatment were backed by the Ministerial Decree N°. 863 of November 4, 2002, the first edition of the Brazilian protocol that established the treatment of hepatitis C with peginterferon alfa in the National Health System [33] and the Ministerial Decree N°. 34 of 28 November 2007, an update of the first ordinance described above [34].

### Treatment

All study participants (n = 216) were retreated with peginterferon alfa-2a (PEG-IFN-2a) at a dose of 180 μg or peginterferon alfa-2b (PEG-IFN-2b) at a dose of 1.5 μg/kg body weight, once a week subcutaneously, associated with an oral dose ribavirin: 1,000 mg/day for patients under 75 kg and 1,250 mg/day, for those weighing over 75 Kg for 48 weeks. The doses of PEG-IFN and ribavirin were adjusted because of adverse effects and as medical advice.

### Effectiveness and safety

A quantitative PCR test was obtained at week 12 of retreatment with the objective of observing a reduction of at least 2 log_10_ in viral load compared to the result of pre-treatment. Some patients also showed the qualitative PCR test at 24 weeks of retreatment, and if the result was positive there should be discontinuation of treatment.

For the efficacy analysis percentage of virological response (VR) and sustained virological response (SVR) were calculated.

We studied the predictive value of various parameters such as gender, age, body mass index, initial viral load, type of response to previous treatment (non-responder and relapsed), grade of hepatic fibrosis and transaminases regarding response to treatment (SVR).

The main safety parameters analyzed from the patients’ reports on monthly interviews were body weight, adverse events and laboratory tests (neutropenia / neutrophils count <750/mm^3^, leucopenia / leukocytes count <1.500/mm^3^ and thrombocytopenia / platelet count <50.000/μL, anemia / hemoglobin level <8.5 g/dL, creatinine, TSH, ALT e AST levels) and total discontinuation due to adverse events. We also analyzed the adverse events that led to discontinuation of treatment.

### Statistical analysis

We performed a descriptive analysis of demographic and clinical variables, and SVR. For quantitative variables we used the mean and standard deviation (symmetric distribution) or median and interquartile range (asymmetric distribution). For the qualitative variables were used as absolute and relative frequencies. The SVR rate was compared in different groups using the chi-square test or Fisher exact test. Follow-up losses were treated as treatment failures.

For comparison of means was applied Student t test for independent samples (eg average age between SVR and failure in SVR). In case of asymmetry, the Mann–Whitney test was used (e.g. median ALT initial between SVR and failure in SVR).

In multivariate analysis, the assessment of factors independently associated with SVR rate was applied to the Poisson regression analysis. The criterion for entering the variable in the model was to produce a p value less than 0.20 in bivariate analysis. We calculated relative risks (RR) and corresponding 95% confidence.

The level of significance was 5% (p ≤ 0.05) and analyses were performed using Statistical Package for Social Sciences (SPSS) version 17.0.

### Ethical aspects

This project was approved by the Ethics Committee of the School of Public Health of Rio Grande do Sul/Brazil, under number 421/08. All subjects signed a consent form in which information was provided on risks and benefits of the drug and confidentiality of data.

## Results

### Patient characteristics

Between September 2003 and March 2009, 216 patients were included in the study. All patients had viral genotype 2 (5.6%) or three (94.4%) and had been treated before for at least 24 weeks with conventional interferon in combination with ribavirin. The demographic, virological and histological characteristics of the sample are presented in Table 1 Out of the 216 patients, 123 (56.9%) were male and mean age between genders was 53.6 ± 9.0 years. More than half had a viral load over 800.000 UI/mL (64.5%), and the mean body mass index (BMI) was 27.5 ± 4.7 kg/m2 among patients.

**Table 1:** Baseline characteristics of patients

**Characteristics**	**Number of patients (n = 216)**
Gender, M/F (%male)	123/93 (56.9)
Age (yrs) (mean ± SD) *	53.6 ± 9.0
Initial Weight (Kg) (mean ± SD)*	76.4 ± 14.0
BMI (Kg/m2) (mean ± SD)*	27.5 ± 4.7
Median Aminotrasferase levels. U/L (range)**	
Baseline Alanine aminotrasferase (ALT)	95 (61–165)
Baseline Aspartate aminotransferase (AST)	84.5 (52–123)
HCV Genotype, n (%)	n = 216
Genotype 2	12 (5.6)
Genotype 3	204 (94.4)
Response to previous treatment, n (%)	n = 216
Non-responders	128 (59.3)
Relapsers	88 (40.7)
Histological diagnosis, n (%)***	n = 196
Fibrosis stage 0 – 2	47 (24.0)
Fibrosis stage 3 – 4	149 (76.0)
Baseline serum HCV RNA (IU/mL), n (%)****	n = 155
< 800.000	55 (35.5)
≥ 800.000	100 (64.5)

*Mean ± standard deviation.

**Median (range percentile 25 – 75).

***Twenty missing (9.3%).

****Sixty-one missing (28.2%).

The completion of the examination of the liver biopsy showed fibrosis and inflammatory activity and results were presented according to the classification of Metavir. Out of the 216 patients retreated it was possible to determine the degree of fibrosis in 196 patients and the inflammatory activity of 170 patients. Forty-seven (24%) had fibrosis grade F0-F2 and 149 patients (76%) had fibrosis F3-F4 (Table 1).

Out of the patients who participated in the study 128 (59.3%) were classified as non-responders and 88 (40.7%) as relapsers to previous therapy with conventional interferon combined with ribavirin (Table 1).

### Safety evaluation

The reasons for treatment interruption are shown in Table 2. In the total sample 51 (23.6%) patients prematurely discontinued treatment. A percentage of 18.5% the withdrawal was due to adverse events, 2.3% for presenting Qualitative PCR positive at week 24 of treatment and 2.8% for other reasons such as no reduction in viral load at 12 weeks of treatment and withdrawal by the patient. The need for dose reduction of peginterferon alfa and/or ribavirin due to laboratory abnormalities (anaemia, neutropenia or thrombocytopenia) occurred in 28 (13%) patients.

**Table 2:** Reasons for discontinuation of treatment

**Reasons for discontinuation**	**Number of patients,n (%)**
Adverse Events	40 (18.5)
No reduction of 2 log viral load at week 12	3 (1.4)
Positive PCR qualitative at week 24	5 (2.3)
Withdrawal of the patients	3 (1.4)
Total discontinuation of treatment	51 (23.6)

Tables 3 and 4 present the adverse events that led to discontinuation of treatment. Laboratory abnormalities were the event that led to more treatment interruption (40%) and thrombocytopenia and leukopenia or neutropenia were more frequent than anemia. The death was the second most frequent event (20%). The decompensated cirrhosis, characterized by encephalopathy and ascites, representing 17.5% of these interruptions. The other events leading to discontinuation were disabling symptoms (fatigue, malaise, diarrhea) (10%), acute renal failure (7.5%), psychiatric disorders (5.0%), psoriasis (2.5%) and diagnosis of hepatocellular carcinoma (2.5%).

**Table 3:** Incidence of adverse events that caused discontinuation of treatment

**Adverse events (n = 40)**	**Incidence of occurrence, n (%)**
Laboratory abnormality	16 (40)
Descompensated cirrhosis (ascites/encephalopathy)	7 (17.5)
Incapacitating symptoms	4 (10)
Acute Renal Failure	3 (7.5)
Psoriasis	1 (2.5)
Diagnosis of Hepatocellular carcinoma	1 (2.5)
Death	8 (20)

**Table 4:** Incidence of major adverse events that caused discontinuation of treatment

**Adverse events**	**Incidence of occurrence, n (%)**
Laboratory abnormality	n = 16
Anaemia	11 (68.7)
Thrombocytopenia/neutropenia	15 (93.7)
Death	n = 8
Acute myocardial infarction	1 (12.5)
Pneumonia and sepsis	2 (25)
Acute respiratory failure	2 (25)
Encephalopathy/ascites	2 (25)
Cause unknown	1 (12.5)

### Viral response and SVR predictive factors

One hundred sixty-five patients (76.4%) completed 48 weeks of retreatment. The overall rate obtained from ETR was 140/216 (64.8%) and from SVR was 88/216 (40.7%). For the group of non-responders patients the ETR rate was 81/128 (63.3%) and the group of relapsed patients the ETR rate was 59/88 (67%), p = 0.671, not showing statistically significant difference between the two groups. In the evaluation of the SVR group of patients non-responders had a rate of 44/128 (34.4%) significantly lower than the SVR rate for the relapsers group 44/88 (50%), p = 0.031(Table 5).

**Table 5:** End-of-treatment and sustained virologic response rates in patients treated to week 48

**End-of-treatment response**	**Patients treated n = 216, n (%)**	p*
End-of-treatment Response at week 48, n (%)	140/216 (64.8%)	
Non-responder to initial treatment	81/128(63.3%)	0.671
Relapsed to initial treatment	59/88 (67%)	
Sustained Virologic Response at week 72, n (%)	88/216 (40.7%)	
Non-responder to initial treatment	44/128 (34.4%)	0.031
Relapsed to initial treatment	44/88 (50%)	

* Significance based on Fisher’s Exact test.

Efficacy data for each group of parameters were analyzed separately in the bivariate analysis of possible predictors versus SVR and are presented in Table 6 The mean age of patients with SVR was 51.3 ± 9.7 years, significantly lower compared with the group of patients who failed to achieve SVR that was 55.2 ± 8.2 years (p = 0.002).

**Table 6:** Bivariate analyse of factors predictive of sustained virologic response (SVR)

**Parameter**	**n**	**SVR**	**Failure SVR**	p*
	**216**	**n = 88**	**n = 128**	
Age (yrs) (mean ± SD)**		51.3 ± 9.7	55.2 ± 8.2	0.002
Gender	216			
Male	123	50 (40.7%)	73 (59.3%)	1.000
Female	93	38 (40.9%)	55 (59.1%)	
BMI (Kg/m2)	143			
< 30	104	41 (39.4%)	63 (60.6%)	0.591
≥ 30	39	18 (46.2%)	21 (53.8%)	
Median Aminotrasferase levels, (U/L)***	215	n = 88	n = 127	
ALT		93 ± 108	97 ± 110	0.144
Histological diagnosis****	196			
Fibrosis stage 0 – 2	47	29 (61.7%)	18 (38.3%)	0.001
Fibrosis stage 3 – 4	149	48 (32.2%)	101(67.8%)	
Baseline serum HCV RNA (IU/mL)*****	155			
< 800.000	55	25 (45.5%)	30 (54.5%)	0.714
≥ 800.000	100	41 (41.0%)	59 (59%)	

* Significance based on qui-quadrado de Pearson test, t-student and Mann–Whitney.

**Mean ± standard deviation.

***Median (interquartile range).

****Twenty missing (9.3%).

*****Sixty-eight missing (31.5%).

*****Sixty-one missing (28,2%).

When analyzed by gender, male patients showed a SVR rate 50/123 (40.7%) females and 38/93 (40.9%), p = 1.000, showing the same response profile. Among patients with initial BMI <30 Kg/m2 or ≥ 30 Kg/m2 SVR rates were 41/104 (39.4%) and 18/39 (46.2%), p = 0.591, respectively, showing equal response between the groups. The mean baseline pre-treatment ALT of patients with SVR was 93 ± 108 U/L, not statistically significant difference if compared with the group of patients who failed to obtain that SVR was 97 ± 110 U/L (p = 0.144). Among patients with histological diagnosis in liver biopsy F0-F2 fibrosis in the SVR rate was 29/47 (61.7%) significantly increased compared with F3-F4 fibrosis where the SVR rate was 48/149 (32.2%), p = 0.001. In assessing the baseline level of serum HCV RNA there was no significant difference between patients with baseline viral load <800.000 UI/mL that showed SVR rate 25/55 (45.5%) compared with a baseline viral load ≥ 800.000 IU/mL where the SVR rate was 41/100 (41%), p = 0.714.

In multivariate analysis, the assessment of factors independently associated with SVR rate was applied to the Poisson regression analysis, using as criteria for variable entry into the model p-value less than 0.20 bivariate analyses (Table 7). Thus, in this study were identified as predictors of SVR age (mean age 51.3 ± 9.4 years, p = 0.005), the type of response to previous treatment (non-responder or relapsed, p = 0.023) and degree fibrosis shown on histological diagnosis (fibrosis F0-F2 or F3-F4 fibrosis, p = 0.004), indicating that patients with less advanced age, relapsed to previous treatment and degree of fibrosis F0-F2 have a better chance of obtaining SVR. For the analysis of variable average initial pretreatment ALT (p = 0.216) the difference was not statistically significant, indicating that this parameter, in this study did not contribute to increase the rate of SVR.

**Table 7:** Multivariate analyse of factors predictive of SVR through regression of poisson

**Parameter**	**RR (IC 95%)**	p*
Age (yrs)	0.98 (0.97-0.99)	0.005
ALT/TGO (U/I)	1.00 (0.99-1.01)	0.216
Histological diagnosis		
Fibrosis stage 0-2	1.68 (1.19-2.37)	0.004
Fibrosis stage 3-4	1.00	
Response to previous treatment		
Non-responders	1.00	
Relapsers	1.46 (1.05-2.02)	0.023

## Discussion

Many patients with chronic hepatitis C have not yet been able to obtain SVR with anti-HCV therapies, and thus become a candidate likely to develop progressive liver disease in the long term, such as cirrhosis or HCC with the possibility of needing liver transplantation [18-20]. Thus, infection in patients with chronic hepatitis C relapsers or non-responders to previous therapy has been an important public health problem and the possibility of an alternative to retreatment has been the focus of clinical investigations.

So, this cohort study was able to assess the effectiveness of retreatment with peginterferon alfa in patients with chronic hepatitis C genotype 2 or 3 in a healthcare environment of the Brazilian public health system. Baseline characteristics such as age, gender, initial body weight, body mass index and baseline ALT and AST, showed no differences from the baseline features presented in several published studies, indicating that the populations for these characteristics were similar.

In our study group of patients non-responders to previous therapy with interferon alfa combined with ribavirin showed an SVR rate of 34.4% when retreated with PEG-IFN alpha-2a or 2b combined with ribavirin for 48 weeks. This result is similar to that described by Sherman et al. who found an SVR rate of 37% in non-responders retreated with PEG-IFN alpha-2a and RBV [30]. In the study of HALT-C Shiffman et al. to assess a sample of 604 patients and all non-responders with advanced fibrosis (METAVIR F3-F4) obtained SVR rate was 18% [21] as well as the study of Carnicer et al. to assess a sample of 35 patients non-responders, 17% with fibrosis F3-F4 and 45.8% with HCV RNA > 850.000 UI/mL showed an SVR rate of only 27.3% [22]. However, some multicenter studies, sponsored by pharmaceutical companies as Parise et al. with PEG-IFN alpha-2a plus RBV [23] and Krawitt EL et al. with PEG-IFN alpha-2b plus RBV [25], has demonstrated higher values in the SVR rates in patients non-responders to prior therapy of 46% and 57%, respectively.

For the group of relapsed patients, in our study we found an SVR rate of 50%. This result is confirmed favorably to the data presented in studies by Mathew et al. who found an SVR rate of 50% of patients relapsed when retreated with PEG-IFN alpha-2b plus RBV [27] as well as the study of Sherman et al. who found an SVR rate of 51% for relapsed patients, genotype 2 and 3 when retreated with PEG-IFN alpha-2a plus RBV [30]. Meanwhile, other authors have presented their studies in SVR rates for patients relapsed to prior therapy, superior to that found in our study, as presented by multicenter studies by Parise et al. PEG-IFN alpha-2a plus RBV [23] and Krawitt E.L et al. PEG-IFN alpha-2b plus RBV [25], SVR rates of 70% and 59%, respectively. Other studies also showed a higher SVR rate than our results, as well as the one presented by Basso et al. to portray patients with recurrent PEG-IFN alpha-2b plus RBV where the SVR rate achieved was 78.6% [32] and Gonçalves Jr et al. who found an SVR rate of 62%, to portray with PEG-IFN alpha-2b plus RBV a sample where only 26% of patients had liver diagnostics F3-F4 and 30% had baseline viral load HCV RNA > 800.000 UI/mL [29].

According to Mitchell L. Shiffman clinical and virological factors may be useful to predict the likelihood of response to retreatment such as: type of response to previous treatment (non-responders or relapsers), race, type of viral genotype, liver disease severity, current alcohol consumption in which higher response rates can be obtained when patients are carefully selected. Therefore, we concluded that retreatment with PEG-IFN and RBV should be reserved for patients relapsed to prior therapy, which had been done with IFN-α monotherapy and in patients with viral genotype 2 and 3 [23].

Based on these previous findings, current studies of retreatment have evaluated the influence of factors predictive of SVR rates. In our study, among the evaluated parameter settings (type of response to previous treatment, age, gender, BMI, ALT, liver fibrosis and serum HCV RNA), only the type of response to previous treatment, age, the initial level of ALT and the degree of hepatic fibrosis, showed statistical significance in achieving SVR.

In the evaluation of factors independently associated with SVR rate was applied Poisson regression analysis and were identified as predictors of SVR: age, be relapsed to previous treatment and present degree of liver fibrosis F0-F2, indicating that these patients are more likely to have SVR. For the analysis of average initial pretreatment ALT variable was not statistically significant, indicating that this parameter in this study did not contribute to increase the rate of SVR.

These findings resemble those reported by Shiffman et al. that showed that in their multivariate regression analysis the following factors: previous treatment with IFN-α monotherapy, virus C genotype 2 and 3, a serum HCV RNA less than 1.5 million IU/mL, an AST: ALT ratio less than 1.0 and absence cirrhosis on liver biopsy as associated with an increased probability of achieving SVR [21].

Our results also are similar to those presented Sherman et al. Direct comparisons are difficult to achieve, since, Sherman et al. stratified the results among the group of relapsed patients and non-responders to initial treatment to assess predictors of SVR: in the group of relapsed patients, the predictors of SVR were HCV genotype 2 and 3, the Caucasian race and a low initial HCV viral load, and in the patients non-responders, were gender, body weight, BMI, degree of hepatic fibrosis and a low initial HCV viral load as predictors of SVR [30].

No association was found between parameters BMI (< or ≥ 30 Kg/m2) and baseline viral load of HCV RNA (< or ≥ 800.000 IU/mL) at the rate of SVR. This lack of association probably could not be established in our study because the sample was lost for the two parameters of 33.8% and 28.2%, respectively. Loss of data collection for these parameters represented a failure in data collection of the study, not allowing the establishment of relationship with SVR. The study of Sherman et al. identified as a predictor of SVR low initial load HCV RNA in both groups of patients as in non-responders relapsed; whereas for this parameter only BMI was identified as a predictor of response in patients non-responders [30]. As the study of Sherman et al. and HALT-C study of Shiffman et al. also found that an initial viral load less than 1.500.000 IU/mL is associated with an increased probability of SVR [21].

Our study showed that patients with less advanced age are more likely to respond to retreatment regimen, since the patients with SVR had a lower mean age (51.3 ± 9.4 years) compared with patients who had failures in SVR with significantly higher mean age (55.2 ± 8.2 years). This result was also observed by Krawitt et al. to identify non-responders that patients aged 40 years or younger have a higher SVR rate, but for the group of relapsed patients, this significance was not found [25].

It is important to note the poor liver profile of these patients’ with high percentage (76% presented F3/F4) with results in liver biopsy can justify the number of interruptions of treatment and deaths from decompensated cirrhosis (ascites and encephalopathy) found in our study. Some effectiveness studies in the literature revealed different percentages described in cirrhotic patients, and international studies with a smaller percentage as observed by Carnicer et al. [22] and Sherman et al. [30] where 20% and 26%, respectively of the samples were composed of patients with cirrhosis. However, in studies in Brazil are found, generally higher percentages of patients with cirrhosis as shown by the studies of Gonçalves et al. [29] and Parise et al. 40% and 33% respectively of their samples composed of patients with cirrhosis [23].

The percentage of discontinuation due to adverse events (18.5%) in our study was slightly different from controlled clinical studies (5-14%) [36] but similar to studies of effectiveness [21,25,27]. This difference in profile is justified by the presence of comorbidities in this population when are conducted effectiveness studies that portray the reality of drug use, without prior selection of the sample. Almost a third of patients eligible to participate in clinical trials is excluded from the criteria established for inclusion and exclusion. Thus, patients with decompensated cirrhosis, hepatitis B, HIV, kidney disease, neuropsychiatric, coronary, cerebrovascular, or hematologic diseases are usually excluded from controlled clinical studies, favoring the non-appearance of adverse events associated with these comorbidities [36].

Eight patients died during the retreatment, representing 20% of interruptions due to adverse events. The causal relationship between death and retreatment has not been established. However, it is remarkable that in controlled clinical studies of effectiveness investigated there are no reports of death during the retreatment [21-32]. The average age of patients who died was 57.3 years and all had degree of liver fibrosis by F4 rating Metavir, characterizing the presence of cirrhosis in the whole population. These criteria may have compromised the continuity of treatment, as well as contributed to the evolutions for the deaths, but since these criteria are included in the general criteria for inclusion of patients candidates for retreatment with alfapeguinterferona + ribavirin in accordance with Ordinance No. 34 of 28 September 2007, the Ministry of Health of Brazil, we could not exclude these patients the possibility for retreatment. For this reason, studies like this, which represent the description of a population treated in real life, without prior sample selection, and was able to demonstrate in their analyzes the importance of establishing predictors of response, should be considered and made public in order to prevent individuals who are at risk of a treatment which will not benefit them, producing individual damage and to the public health system.

## Conclusions

The results obtained in our sample suggest that there are benefits in portraying patients who failed therapy with interferon alfa and ribavirin, demonstrating that acceptable response rates, but not ideal yet, can be achieved in clinical practice. Patients non- responders to previous therapy had an SVR rate of 34.4% while relapsed patients a rate of 50% was obtained. The less advanced age, to be relapsing to previous treatment and present with liver biopsy fibrosis (F0-F2 Metavir) were identified as best predictors of SVR.

Concurrently with these findings the data safety profile of this treatment should be considered, since 18.5% of the reasons for prematurely discontinuation of treatment were due to adverse events, being the most frequent laboratory abnormalities (40%) and death (20%).

So, considering the severity of this clinical and epidemiological condition, it is necessary a careful selection of patients to retreatment, in order to accommodate patients who meet the criteria of predictive factors of SVR, in order to not to expose individuals to the risk of treatment that will not be benefited, producing individual damage and to the public health system.

## Abbreviations

ALT: Alanine aminotransferase; AST: Aspartate aminotransferase; BMI: Body mass index; CAMID: Center for Application and Monitoring of Injectable Drugs; CVH-PTG: Chronic Viral Hepatitis C Therapeutic Guideline; FDA: Food and Drug Administration; HCV: Hepatitis C virus; IFN: Interferon; IFN-2a: Interferon alfa-2a; IFN-2b: Interferon alfa-2b; PCR: Polymerase Chain Reaction; PEG-IFN 2a: Peginterferon alfa-2a; PEG-IFN 2b: Peginterferon alfa-2b; PSH: Parthenon Sanatorium Hospital; RBV: Ribavirin; RFT: Percentage of virological response; RR: Relative risks; SPSS: Statistical Package for Social Sciences; SVR: Sustained virological response; VR: Virological response.

## Competing interests

The authors declare that they have no competing interests.

## Authors’ contributions

SA carried out the interviews pharmacotherapeutic monitoring, developed the database, performed the statistical analysis and drafted the manuscript. KA carried out the interviews pharmacotherapeutic monitoring and performed the statistical analysis. CG participated in the design of the study. PP conceived of the study, and participated in its design and coordination and helped to draft the manuscript. All authors read and approved the final manuscript.

## Pre-publication history

The pre-publication history for this paper can be accessed here:

http://www.biomedcentral.com/1471-2334/12/377/prepub
